# In Our Own Words: The Complex Sensory Experiences of Autistic Adults

**DOI:** 10.1007/s10803-021-05186-3

**Published:** 2021-07-13

**Authors:** K. MacLennan, S. O’Brien, T. Tavassoli

**Affiliations:** School of Psychology and Clinical Language Sciences, University of Reading, Earley, Reading, RG66BZ UK

**Keywords:** Autism, Autistic, Adult, Sensory, Participatory, Qualitative

## Abstract

Autistic adults commonly experience sensory reactivity differences. Sensory hyperreactivity is frequently researched, whilst hyporeactivity and seeking, and experiences across domains, e.g., vision, are often neglected. Therefore, we aimed to understand more about the sensory experiences of autistic adults. We conducted a mixed-methods study, co-produced with stakeholders; recruiting 49 autistic adults who completed an online survey. Firstly, quantitative results and content analysis enhanced our understanding of sensory input/contexts associated with sensory hyperreactivity, hyporeactivity, and seeking across modalities. Secondly, thematic analysis developed themes relating to ‘Outcomes’, ‘Control’, ‘Tolerance and management’, and ‘The role of other people’, informing a theoretical model of sensory reactivity differences in autistic adults. These findings have implications for support services and improving quality of life for autistic adults.

**Supplementary Information:**

The online version contains supplementary material available at 10.1007/s10803-021-05186-3.

## Introduction

Autism Spectrum Conditions (ASC) are neurodevelopmental conditions typically characterised by social communication differences, and restricted and repetitive interests and/or behaviours (RRBs) (DSM-5 American Psychiatric Association, 2013). Sensory reactivity differences, a sub-criterion under RRBs, are suggested to be present in up to 94% of autistic adults^1^ (Crane et al., 2009). Sensory reactivity differences can occur across multiple sensory domains, such as vision or touch, and are characterised by hyperreactivity, hyporeactivity, and sensory seeking (DSM-5 American Psychiatric Association, 2013). Individuals who are sensory hyperreactive often experience sensory input more intensely compared to others, and may find it painful, dysregulating, or overwhelming (Lane, 2002). Individuals who are sensory hyporeactive often have a delayed response, or may not notice, sensory input, and it may be experienced by individuals after periods of hyperreactivity (Lane, 2002; Liss et al., 2006). Individuals who are sensory seeking often engage with sensory input repeatedly and/or for sustained periods of time, and it may be a stimulatory or regulatory strategy associated with RRBs, sensory hyperreactivity and/or hyporeactivity (Lidstone et al., 2014; Pellicano et al., 2013; Schulz & Stevenson, 2019). Sensory experiences can be complex and although some sensory experiences can be enjoyable for individuals, other experiences can be very distressing, impacting on quality of life and correlating with mental health conditions, such as anxiety and depression (Carpenter et al., 2018; Elwin et al., 2012; Forsyth & Trevarrow, 2018; Green et al., 2012; Hwang, 2019; MacLennan et al., 2020, 2021; Rossow et al., 2021). It is therefore unsurprising that difficulty with sensory input has been suggested to be a barrier for autistic adults engaging in spaces, both public and occupational (Amos et al., 2019). Thus, understanding the complexities of sensory experiences has important implications for autistic people’s physical and mental wellness, social inclusion, and future prospects.

Previous qualitative findings have shown that autistic adults experience sensory hyperreactivity across multiple domains, such as finding sounds too loud and painful, becoming distracted by nearby conversations, as well as having aversions to competing sounds, bright colours, bright or flickering lights, light touch, clothing, overpowering scents, and food tastes and textures (Chamak et al., 2008; Jones et al., 2003; Robertson & Simmons, 2015). Additionally, sensory hyperreactivity can result in autistic adults feeling overwhelmed, and this can be exacerbated when experiencing heightened stress and depleted energy levels (Chamak et al., 2008; Robertson & Simmons, 2015; Smith & Sharp, 2013). This can create a vicious cycle, where sensory hyperreactivity creates stress, which amplifies sensory hyperreactivity, leading to more stress (Smith & Sharp, 2013).

However, existing research has provided much less insight into experiences of sensory hyporeactivity and seeking. Recent research has shown that although sensory hyporeactivity may be less pronounced in adulthood, it is still present (Hwang et al., 2019). Qualitative research has suggested that autistic adults experience sensory hyporeactivity to pain, hunger, temperatures, scents, flavours, as well as certain sounds (Chamak et al., 2008; Elwin et al., 2012). As for sensory seeking, research has thought that it may be more prominent in childhood compared to adulthood (Kern et al., 2007). However, qualitative research has suggested that autistic adults seek out enjoyable and soothing sensory perceptual experiences, such as favourite music, and feeling certain textures such as cold, smooth surfaces (Jones et al., 2003; Robertson & Simmons, 2015). But, due to the underrepresentation of sensory hyporeactivity and seeking in research, there is an imperative need to understand more about autistic adults’ experiences of these types of sensory reactivity.

Despite evidence suggesting that sensory reactivity differences persist into adulthood (Crane et al., 2009), the sensory experiences of autistic adults have been under-represented in research. Furthermore, sensory hyperreactivity is often a key focus of research, despite evidence that autistic adults experience varying patterns of sensory reactivity differences (Crane et al., 2009). Research has yet to comprehensively identify sensory input that is related to sensory reactivity differences in autistic adults, especially input relating to hyporeactivity and seeking, across modalities (e.g., vision, touch). Furthermore, it is yet to examine autistic adults’ experiences of these sensory reactivity differences, also involving autistic individuals in the research process. Therefore, the present mixed-methods study aimed to elucidate the complex sensory experiences of autistic adults using a novel online survey approach. Firstly, we sought to understand more about elements of the sensory environment relating to sensory hyperreactivity, hyporeactivity, and seeking across sensory domains, such as visual, auditory, and tactile. Secondly, we sought to understand more about autistic adults’ sensory experiences related to these sensory reactivity differences, to develop a theoretical model reflecting these experiences. Importantly, this research was co-produced with autistic adults following a participatory research framework (Pellicano et al., 2013), to ensure the research was shaped by autistic individuals and relevant and consistent with their values.

## Methods

### Design

This study adopted a mixed-methods design, using both quantitative and qualitative approaches, as well as principles of a participatory research framework (Pellicano et al., 2013). The research team included a doctoral researcher (KM) and an associate professor (TT), who specialise in autism and sensory research, as well as an autistic researcher (SOB), with lived experience and autism research expertise. Feedback from members of the autistic community, external to the research team, was sought at key stages of the projects to improve accessibility, such as the design of recruitment materials, the information sheet, and the questionnaire, as well as an insight group with four autistic adults to discuss the interpretation of results.

The data was collected using an online mixed-methods survey, including both closed and open-ended questions. Collecting the qualitative data using an online survey was chosen over other methods, such as focus groups or interviews. This is because qualitative surveys have the advantage of being able to capture what is important to participants using their own language and terminology (Frith, 2000), and this can easily be combined with the simultaneous collection of quantitative data online. Qualitative surveys are argued to achieve the depth and richness needed for qualitative research through a ‘wide-angle lens’; gaining perspectives and experiences from a diverse range of voices from widely geographically dispersed populations (Braun et al., 2017; Toerien & Wilkinson, 2004). This is especially important when the group of interest are large or diverse (Braun et al., 2017), which is effective for research with autistic populations, due to their heterogeneous nature. Online methods also improve the feeling of anonymity, reducing social desirability, and also lessen the burden of participation as the survey can be completed flexibly in a time, pace, and place that suits the participant (Braun et al., 2020).

### Participants

The data of 49 autistic adults was included in the analysis, age range 20–55 years (mean = 34.5, SD = 10.6), although 14 adults chose not to disclose their age (see Table 1). All participants self-reported that they had been clinically diagnosed as autistic. None of the participants had significant hearing or visual impairments that could confound their sensory reactivity. Specific data on socioeconomic status and educational attainment levels were not obtained for this study.

**Table 1:** Demographic characteristics of participants

	*N*
Gender	
Male	11 (22.5%)
Female	33 (67.5%)
Non-binary	5 (10.0%)
Ethnicity	
White British	19 (38.8%)
White European	3 (6.1%)
White British/American	1 (2.0%)
White non-specified	21 (43.0%)
Ashkenazi Jewish	1 (2.0%)
Latina	1 (2.0%)
Not specified	3 (6.1%)
Self-reported clinical diagnoses	
ASC	49 (100%)
Anxiety	21 (43.0%)
Depression	14 (28.6%)
ADHD	9 (18.4%)
Bipolar disorder	4 (8.2%)
Anorexia nervosa	4 (8.2%)
Intellectual disability	2 (4.1%)
Borderline personality disorder	1 (2.0%)
No diagnoses in addition to ASC	14 (28.6%)

*ASC* autism spectrum condition; *ADHD* attention deficit hyperactivity disorder

**Fig. 1:**
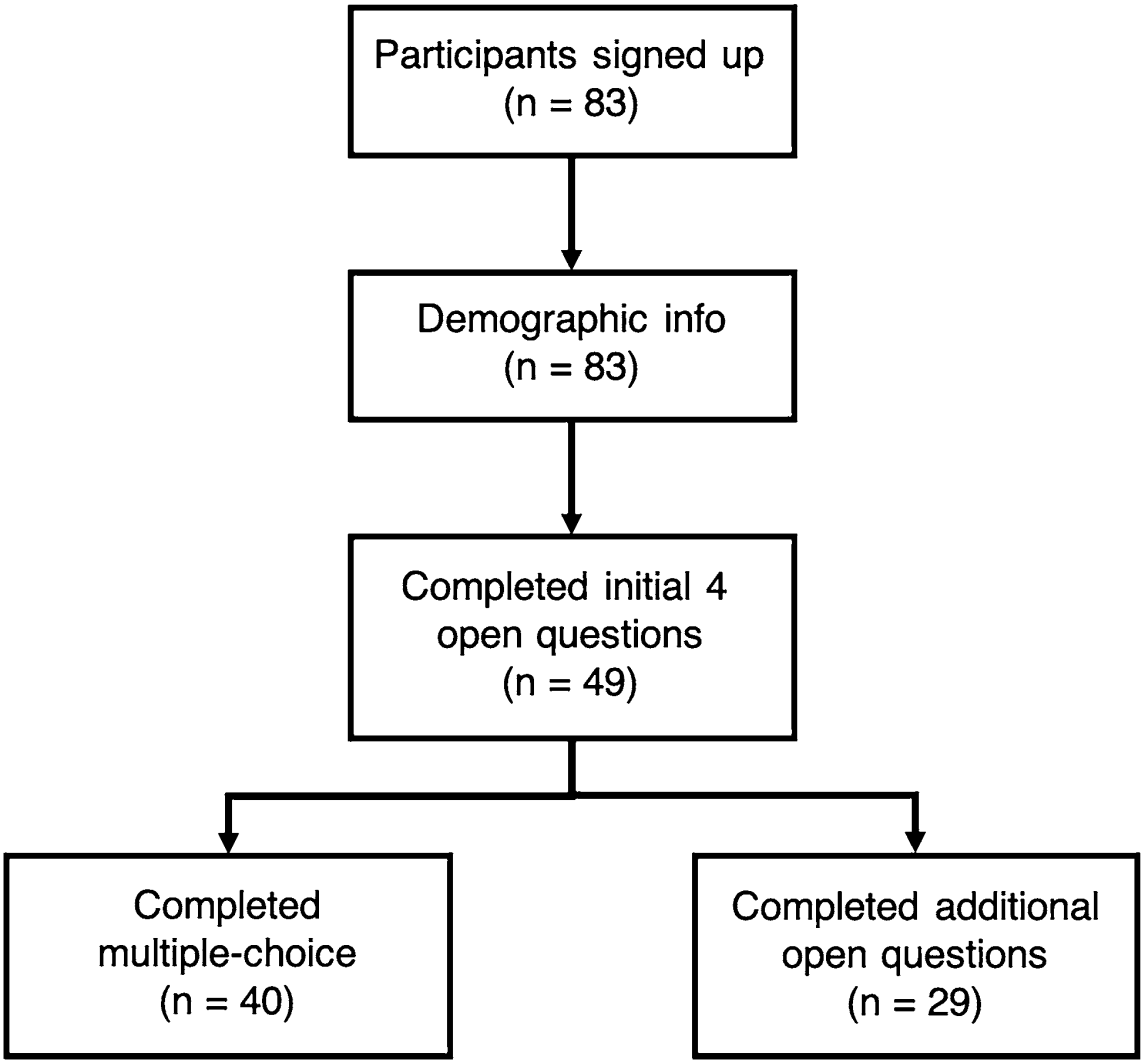
Flow diagram showing the number (n) of participants included at each stage

Eighty-three participants were originally recruited for the study; however, 34 participants did not proceed with the study after completing the demographic information and were excluded. Out of the 49 participants that were included in the study, 40 provided responses to the multiple-choice questions, 49 completed the first section of open questions, and 29 completed the full qualitative questionnaire (Fig. 1). The autistic adults were recruited via the Centre for Autism, University of Reading, participant database, and through Facebook and Twitter social media posts. All participants provided informed consent online before commencing with the study. Ethical approval was granted prior to the commencement of this study by the University of Reading Ethics Committee.

### Measures

We developed an online questionnaire including demographic questions followed by 34 questions to capture information about the sensory experiences of autistic adults; 28 were open ended and six were multiple choice. We also provided descriptions of sensory hyperreactivity, hyporeactivity and seeking, and these terms were used throughout the questionnaire. The questionnaire was designed to capture participants’ general experiences of being sensory hyperreactive, hyporeactive, seeking and/or neutral to the sensory environment, as well as for each modality; visual, auditory, tactile, interoception, gustatory, and olfactory. The initial 4 questions asked broadly about (1) sensory hyperreactivity, (2) sensory hyporeactivity, (3) sensory seeking, and (4) neutral sensory experiences, e.g., “Describe your experience of being sensory hyperreactive”. Followed by questions asking about sensory experiences relating to sensory modalities, e.g., “Describe your experiences of being hyperreactive to the visual environment.” Additionally, the questionnaire included six multiple-choice questions that asked participants if they experienced sensory reactivity differences (hyperreactivity, hyporeactivity, seeking, and/or neutral) to pre-defined aspects of the sensory environment in each modality (e.g., visual: bright lights), and an ‘other’ option for them to add their own. These stimuli were identified from existing literature and tools (Brown et al., 2001; Schoen et al., 2008), and from a group of autistic adults during the development of the questionnaire. These closed questions were included to provide examples to assist in answering the qualitative questions and allowed participants who may struggle with open questions to still share their sensory experiences.

### Procedure

Autistic adults were invited to take part in an online questionnaire about their sensory experiences. Participants were able to read the study information and then provide informed consent via an online form. It was also highlighted that although the answers to questions had to be their own, they should seek support with completing the questionnaire if required. Participants then completed the survey, which was anticipated to take between 15 and 60 min depending on the depth of information provided.

### Analysis

To address our research aims, we used a mixed-methods analysis approach. The data was analysed using Nvivo (Castleberry, 2012) primarily by one of the researchers (KM), but the development and interpretation of categories, codes and themes were discussed and confirmed by all members of the research team (SOB and TT), as well as the four autistic adults from the insight group.

To elucidate the elements of the sensory environment associated with sensory reactivity differences, including hyperreactivity, hyporeactivity, and seeking for each modality, we firstly analysed the multiple-choice data by calculating the percentage of participants that identified as having these experiences in each modality. Secondly, we analysed the qualitative data using content analysis, which is an objective systematic way of quantifying and describing data (Elo & Kyngäs, 2008; Krippendorff, 2018). The categories contain words and phrases that share meaning (Cavanagh, 1997). To achieve our aims, deductive, a priori coding was adopted to predetermine the categories of sensory reactivity differences and domain, however we adopted an unconstrained matrix where we used an inductive process to identify sub-categories of common stimuli and contexts within these bounds (Elo & Kyngäs, 2008).

Then, to understand more about autistic adults’ sensory experiences, we analysed the data using thematic analysis; identifying patterns of meaning in the data (Braun & Clarke, 2006). We took an inductive approach so our analysis was driven by the data rather than preconceived coding or perceptions, and we adopted an iterative framework to develop meaning from the data (Srivastava & Hopwood, 2009). Thus, our approach was a continuous and deeply reflexive process, which recognised that categories and themes do not emerge on their own but are driven by what we as researchers aimed to know and how we interpreted the data. The framework proposes three questions as reference points, (1) What are the data telling me? (2) What is it I want to know? (3) What is the dialectical relationship between what the data are telling me and what I want to know? Through this cyclical approach, categories and themes were reflexively revised and refined, considering researcher biases, and incorporating insights from the analysis process and from checks with the research team and members of the autistic community. Once the final themes were developed, these were then adapted into a theoretical model. The importance of highlighting the interconnected nature of the themes was emphasised by the autistic adults in the feedback group. Therefore, the model is informed by the themes and provides enhanced representation of the lived experience of sensory reactivity differences for autistic adults.

## Results

### Quantitative Analysis

**Fig. 2:**
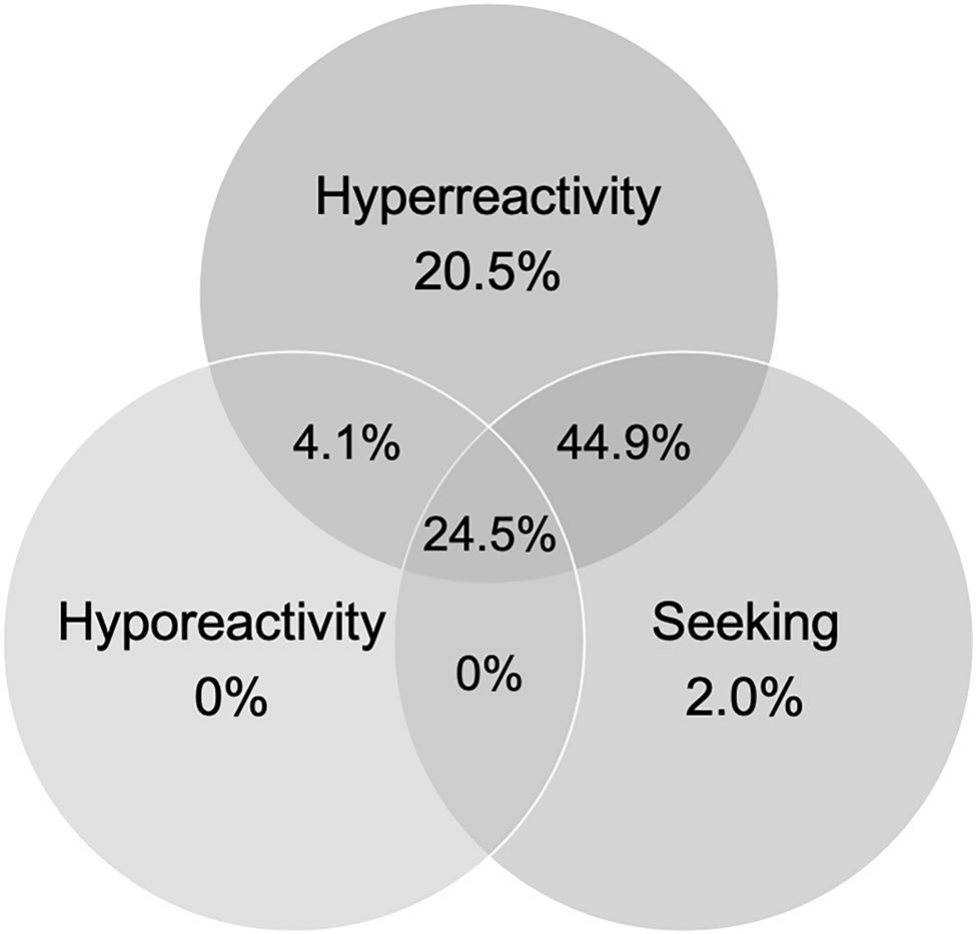
Depicting the percentage of participants (n = 49) self-identifying as experiencing only sensory hyperreactivity, hyporeactivity, or seeking, or experiencing hyperreactivity and hyporeactivity, hyperreactivity and seeking, or hyporeactivity and seeking, or experiencing all three

Responses to the multiple-choice questions found that 93.9% percent of the autistic adults identified as experiencing sensory hyperreactivity, 28.6% identified as experiencing sensory hyporeactivity, and 41.4% identified as experiencing sensory seeking (Fig. 2; see Table 2 for sensory input/contexts associated with sensory reactivity differences in each modality). Furthermore, 22.5% identified as experiencing only either sensory hyperreactivity, hyporeactivity, or seeking (20.5% hyperreactivity; 0% hyporeactivity; 2.0% seeking), 49.0% identified as experiencing 2 of these (4.1% hyperreactivity and hyporeactivity; 44.9% hyperreactivity and seeking; 0% hyporeactivity and seeking), and 24.5% identified as experiencing all 3. Although all participants reported qualitative experiences of sensory reactivity differences, 4.1% of the autistic adults identified as having no sensory reactivity differences.

**Table 2:** Summary of quantitative analysis

	Percentage %			
	Hyperreactive	Hyporeactive	Seeking	Neutral
Overall				
Yes	93.9	28.6	41.4	–
No	6.1	71.4	28.6	–
Visual				
Bright lights	75.0	5.0	0.0	17.5
Flashing lights	75.0	0.0	0.0	22.5
Bright colours	27.5	0.0	30.0	40.0
Low contrast images	7.5	7.5	7.5	77.5
Patterns	35.0	2.5	35.0	30.0
Auditory				
Lots of conversations	82.5	6.0	2.5	2.5
Shopping centres	26.0	5.0	5.0	20.0
Public transport	70.0	7.5	0.0	17.5
Loud noises	87.5	5.0	2.5	2.5
Ambient noises	40.0	7.5	10.0	47.5
High pitch noises	77.5	2.5	2.5	12.5
Music	75.0	0.0	60.0	15.0
Tactile				
Pressure differences	47.5	5.0	32.5	17.5
Clothing,	75.0	0.0	15.0	15.0
Different textures	62.5	0.0	73.5	12.5
Interoception				
Cold temperatures	42.5	12.5	7.5	27.5
Hot temperatures	55.0	15.0	2.5	20.0
Changes in the weather	47.5	10.0	2.5	35.0
Physical pain	35.0	30.0	5.0	25.0
Gustatory				
Spicy food	37.5	10.0	30.0	25.0
Food temperatures	32.5	5.0	10.0	42.5
Food textures	65.0	2.5	17.5	22.5
Favourite foods	22.5	5.0	37.5	32.5
Memorable foods	20.0	2.5	25.0	45.0
Chewing gum	10.0	0.0	20.0	57.5
Olfactory				
Strong scents	65.0	10.0	10.0	15.0
Perfumes	60.0	10.0	12.5	12.5
Pollution	45.0	7.5	0.0	40.0
Flowers	27.5	15.0	20.0	30.0
Fresh air	12.5	12.5	35.0	40.0

Percentage of participants in the multiple-choice responses who identified experiencing sensory reactivity differences to sensory stimuli for hyperreactivity, hyporeactivity and seeking, and in each modality (n = 40)

### Sensory Hyperreactivity

Responses to the multiple-choice items showed that autistic adults identified they are commonly hyperreactive to bright and flashing lights (75%), loud noises (87.5%), lots of conversations (82.5%), high-pitch noises (77.5%), music (75%), public transport sounds (70%), clothing (75%), different textures (62.5%), hot temperatures (55%), food textures (65%), strong scents (65%), and the scent of perfume (60%).

### Sensory Hyporeactivity

Overall, in the multiple-choice items, sensory hyporeactivity was minimally selected as being experienced across domains. The most endorsed hyporeactive experiences were in the interoceptive domain, including being hyporeactive to physical pain (30%), and hot (15%) and cold (12.5%) temperatures.

### Sensory Seeking

Responses to the multiple-choice items showed that autistic adults identified they most commonly seek out music (60%), and different textures (73.5%). Other seeking experiences that some of the autistic adults endorsed included looking at patterns (35%) and bright colours (30%), feeling pressure differences (32.5%), favourite (37.5%) or spicy food flavours (30%), and the scent of fresh air (35%).

### Neutral Sensory Experiences

Responses to multiple-choice items found many of the autistic adults did not have any sensory reactivity differences associated with low contrast images (77.5%) or chewing gum (57.5%). Some of the autistic adults also identified having no sensory reactivity differences associated with looking at bright colours (40%) or patterns (30%), ambient noise (47.5%), memorable (45%) or favourite (32.5%) foods, or the scent of fresh air (40%), pollution (40%), or flowers (30%).

### Content Analysis

The qualitative data was then analysed using content analysis to create categories of sensory input/contexts that related to sensory reactivity in each modality. Table 3 shows sensory input/contexts associated with sensory reactivity differences in each modality. Example quotes for these categories can be found in the supplementary materials.

**Table 3:** Summary of content analysis

	Hyperreactive	Hyporeactive	Seeking
Visual			
Bright lights (e.g., artificial, sunlight)	30	–	–
Flashing lights	12	–	–
Cluttered/busy environments	13	–	–
Bright colours	6	–	10
Patterns	5	–	9
Motion/moving objects	5	–	5
Visual search (e.g., slow to see dangers or changes)	–	7	–
Complex images (e.g., nature scenes, artwork)	–	–	9
Ambient lights	–	–	6
Auditory			
Loud and/or unexpected sounds	28	–	–
Busy/chaotic auditory environments	19	–	–
Background noise	18	–	–
Sounds others cannot hear	10	–	–
High pitched noises	6	–	–
Out of tune sounds	3	–	–
Repetitive sounds	3	–	–
No response when distracted/focussed	–	10	–
Own music	–	–	26
Ambient sounds (e.g., fans or engines humming)	–	–	3
Tactile			
Light and/or unexpected touch from people	29	–	–
Clothing fabrics and labels	24	–	–
Tight clothing	9	–	–
Environmental textures (e.g., carpet, blankets, feathers)	9	–	18
Wet/greasy textures (e.g., rain, sweat, lotions)	8	–	–
Pressure (e.g., tight clothing, tight hugs)	–	–	23
Hot or cold items/surfaces	–	–	10
Interoception			
Temperature extremes	17	11	–
Pain	4	15	–
Body signals (e.g., hunger, needing the toilet)	–	4	–
Gustatory			
Food tastes	18	8	24
Food textures	16	–	5
Olfactory			
Strong odours	31	–	–
Perfumes	12	–	5
Food odours	9	–	7
Scented products	8	–	6
People and animals	6	–	4
Dirty home odours (e.g., bins)	6	–	–
Cigarette smoke	5	–	–
Pollution	4	–	–
Subtle odours	–	9	–

Number of participants who reported experiences relating to sensory hyperreactivity, hyporeactivity, and seeking, in each modality (n = 49)

### Sensory Hyperreactivity

Content analysis found that the autistic adults commonly experience hyperreactivity to bright lights, both artificial and to sunlight, as well as to flashing lights, and busy, cluttered environments. Many also reported being hyperreactive to bright colours and patterns, and motion or moving objects. In the auditory domain, hyperreactivity to loud and/or unexpected sounds was the most reported, such as sirens, alarms, and dogs barking. But also, being hyperreactive in response to busy/chaotic auditory environments and to background noise, especially situations with multiple conversations, were commonly reported. In the tactile domain, many of the autistic adults reported being hyperreactive to touch from other people, especially when it is light or unexpected, as well as to different fabric textures and labels in clothing, being unable to wear some clothing because of this. Some of the autistic adults also reported being hyperreactive to tight clothing, and certain rough textures, such as carpets and feathers, and wet/greasy textures, such as sweat or lotions. Regarding interception, many of the autistic adults reported being hyperreactive to temperature extremes; being too hot or too cold. As for Olfactory, many of the autistic adults reported a range of individualistic food aversions and dietary restrictions due to being hyperreactive to certain food tastes and textures. Lastly, many of the autistic adults reported being hyperreactive to what they referred to as *strong odours*, as well as perfume, scented products, or food odours, which they often find unbearable and could result in them feel nauseated. Some of the autistic adults also reported being hyperreactive to people or animal smells, as well as dirty home odours such as bins/trash.

### Sensory Hyporeactivity

Experiences of sensory hyporeactivity were less commonly reported. However, content analysis found that some of the autistic adults reported they were hyporeactive to visual search or environmental changes, such as having difficulty findings an item they’re looking for and being slow to notice changes or danger in their environment. Additionally, some of the autistic adults described experiences of auditory hyporeactivity in instances when they are hyper-focussed or concentrating on a task. Furthermore, many of the autistic adults reported their experiences of being hyporeactive to pain and consider themselves to have a high pain threshold compared to others. Additionally, the autistic adults also described their experiences of being hyporeactive to temperature and may be slow to notice if they feel too hot or cold, or if they touch something that is very hot or cold. Lastly, some of the autistic adults reported that they can struggle to smell subtle scents that other people report to have noticed.

### Sensory Seeking

The content analysis found that many of the autistic adults described how they seek out to look at bright colours and patterns for extended periods of time, as well as complex images, such as scenes of nature or artwork. A few also reported that they seek out motion or moving objects, as well as ambient lighting. As for the auditory domain, the autistic adults commonly reported that they seek out music related to their individual music tastes and will often listen to the same song repeatedly. Regarding the tactile domain, many of the autistic adults described how they seek out pressure, such as by wearing tight clothing and getting tight hugs from trusted people, as well as seeking out certain textures, such as those that are soft or fluffy. Additionally, some of the autistic adults seek out the feeling of warm or cold surfaces on their skin. Relating to the gustatory domain, many of the autistic adults reported that they seek out certain food tastes and textures and would sometimes fixate on certain foods. Lastly, many of the autistic adults described how they continually seek out odours that they like, such as food scents, perfume, scented products, and aromatherapy.

### Thematic Analysis

The iterative approach to thematic analysis led to the development of four themes with 11 sub-themes, that related to sensory reactivity in autistic adults: “Outcomes”, “Tolerance and management”, “Control”, and “The role of other people” (Table 4).

**Table 4:** Summary of main themes and sub-themes developed from thematic analysis relating to sensory reactivity in autistic adults

Main theme	Sub-themes
Outcomes	Physical outcomes and responses Feeling overwhelmed and disengaging Mental health
Control	A desire for control and predictability Difficulty with self-control
Tolerance and management	Moderated by mood Soothing sensory input Avoidance Adaptation
The role of other people	Understanding Support

### Outcomes

Many of the autistic adults reported the impacts of having sensory reactivity differences. They described how sensory experiences can negatively impact their mental health, highlighting links to anxiety, self-harm, and eating disorders. They also reported negative physical responses in response to aversive aspects of the sensory environment, and how these could affect their physical health. The autistic adults also described how sensory stimuli and environments can become overwhelming, which can make them feel like they have disengaged or ‘shutdown’.

#### Mental Health

Several of the autistic adults reported the impact that sensory experiences can have on their mental health. Difficulties with sensory input was described to impact mood, causing stress and agitation:

*“Bright lights such as ceiling lights are unbearable and make me feel very stressed.”* SE007

Many of the autistic adults also reported that aversive sensory experiences were a cause for anxiety. They described how a range of experiences across modalities could trigger anxiety, such as loud sounds, unexpected touch, bright or flickering lights, disliked food tastes and textures, and strong scents:

“When we had issues with our sewage system that persisted for a couple of weeks the constant smell made me agitated and anxious to the point I was experiencing suicidal ideation.” SE016

Additionally, some of the participants reported links between sensory seeking experiences and mental health conditions, for instance, self-harming being described as a form of sensory seeking, and strong liking for certain foods being related to eating disorders:

“I was bulimic for a number of years. This was a form of sensory seeking—I always binged on sweet foods and unlike most bulimics was always very present as I was eating the food.” SE016

#### Physical Outcomes and Responses

Sensory experiences were reported to trigger a range of physical responses that can be intense and overwhelming. Sensory hyperreactivity is often experienced as physical pain, for instance due to sudden loud sounds or unexpected touch, and can induce headaches and nausea, for instance because of strong scents becoming overpowering:

“Smelling a strong smell is like being tortured, time stops and I'm nearly sick.” SE008

“I'm easily startled by sound or touch, sounds physically hurt me.”SE017

Additionally, difficulty with sensory stimuli had implications for physical health, such as auditory hyperreactivity impacting sleep, restrictive or repetitive eating due to difficulties with taste leading to nutritional imbalances, or medical difficulties due to being hyporeactive to pain:

“I think over the years I learned to dissociate from pain and/or found it difficult to recognise or describe what my pain was. This led to many traumatic medical situations where I couldn't say that I was in pain...” SE038

#### Feeling Overwhelmed and Disengaged

Feeling overwhelmed due to sensory input was commonly reported by the autistic adults. Many described overwhelm arising from multi-sensory environments or complex and intense environments. A few of the participants also conveyed that being overwhelmed manifests in disengagement or ‘shutdown’, and they are no longer able to tolerate the sensory environment:

“I can become overwhelmed in busy, crowded places. Often in these situations I will feel like everything around me is moving faster and feel a kind of disconnect (maybe even dissociation?) from it all.” SE03

“… all the loud environmental noise (which may seem like nothing to most people—air conditioning humming, projector buzzing, lights buzzing, plates clinking in a restaurant kitchen), can drive me into a shutdown. And then every sound is utterly overwhelming.” SE040

### Tolerance and Management

The majority of the autistic adults reported a range of adaptive and maladaptive strategies that they use to cope with their sensory reactivity differences and the sensory environment. Many of the autistic adults described how they avoid aversive sensory stimuli or environments they struggle to cope with. However, they also described adaptations they make to be able to cope with sensory input in certain situations. Many of the adults also described how they seek soothing sensory input as a coping strategy, due to the calming effects they experience. However, the autistic adults also described how their ability to cope with sensory input is moderated by their mood, such as when feeling more relaxed, or tired and stressed.

#### Avoidance

Many of the autistic adults described how they avoid sensory stimuli and environments as a coping strategy. Avoidance was often described as the need to ‘run away’ and escape from aversive sensory input when it became too difficult to cope with it. Not being able to escape from aversive sensory input or situations was often reported to be distressing. Some of the autistic adults mentioned certain environments they will avoid for sensory reasons:

“Not being able to escape with summer becomes a big anxiety thing for me—most other negative sensory things can be fixed by leaving the room or going home, this can't, so it just becomes a constant background factor…” SE047

“[I] never go into supermarkets because of visual overload…” SE048

Avoidance was also reported in the form of physical blocking behaviours, such as covering eyes, ear, or nose, or closing eyes or holding breath, in response to visual, auditory, or olfactory stimuli that are difficult to cope with:

“In a crowded place I need to put my hands over my ears. I feel sick and can't focus on anything.” SE027

#### Adaptations

Many of the autistic adults reported different ways they have had to adapt to be able to cope with sensory input in different situations or environments. Several of the adults described tools and strategies that help with their ability to cope, such as using sunglasses to lessen the effects of bright lights or using earplugs or headphones to lessen the impact of loud or busy sound environments, or altering their environments to cope with their sensory difficulties:

“Going to grocery-store is the worst. The lights are always very bright and there are so many details to see. I cannot go in there without sunglasses and a baseball cap.” SE032

“I am very sensitive to all noise... I turn off everything possible and spend time in my quiet bedroom when life gets too noisy.” SE029

Some of the autistic adults also talked about how their sensory reactivity to certain input has adapted over time, and this has meant they are better able to tolerate some sensory input that they previously struggled with:

“I have only been able to tolerate multi-textured food in the past 10 years and still struggle with fruits such as apples as they have an unpredictable texture” SE007

#### Soothing Sensory Input

The majority of the autistic adults reported that they seek soothing sensory input as a way to cope with distressing situations or as a calming strategy when they are distressed. Some of the autistic adults described having ‘toolkits’ of sensory strategies that they could engage with when feeling distressed:

“I always keep a fleecy blanket in my bag to wrap myself tightly in when in distress or just to hold and feel, I lie on my tummy on the floor and ask my husband (over twice my weight) to lie on top of me, love the warm weight of my pet guinea pigs.” SE010

Enjoyable sensory experiences were also reported to be helpful in overshadowing aversive sensory input, for instance listening to music in headphones when in challenging noisy environments or feeling pleasant textures to detract from other input:

“I seek comfort in small things (such as nice textures, I currently have a coin that I keep in my pocket to hold when I'm nervous) so that I can filter the sensory environment. It helps distract me from what is happening around me so that when I seek that input, I can avoid more intense or unpleasant inputs.” SE018

#### Moderated by Mood

Several of the autistic adults reported that the extent of their sensory reactivity can depend on their mood and that this can change whether they are hyperreactive or neutrally reactive to certain sensory input. They described how their tolerance for sensory stimuli, such as sounds, touch, or bright lights, can be better if they are feeling more relaxed or rested. But equally, sensory experiences can be more distressing and aversive if already stressed and tired:

“Sometimes if I'm relaxed things seem to be neutral. If I've slept well, and have had a relaxed day, I can tolerate most noises and lights well.” SE015

“When under stress I am hyperreactive to sound. When not stressed, loud environments make me very tired but I only experience them as in-the-moment unpleasant when stressed or tired.” SE042

### Control

Many of the autistic adults reported how control was related to their sensory reactivity and sensory experiences. A desire for predictability and control over sensory stimuli was commonly described to effect how sensory stimuli is experienced. Similarly, the level of control over the sensory input and intensity of the input can affect whether it is perceived as an enjoyable or aversive experience.

#### A Desire for Control and Predictability

Frequently reported by the autistic adults was a difficulty with unpredictable sensory stimuli. They described how unexpected sensory events can be distressing, such as a sudden siren or being touched by someone. But also, sensory stimuli that is not always the same can be challenging as the experience can be unpredictable:

“Sirens and doorbells make me scared. I cannot concentrate if the noise around me is unpredictable.” SE021

“Fruit is the hardest for me because the tastes are so variable, such as one apple to the next.” SE007

The autistic adults also reported how if they are in control of the intensity, then certain sensory experiences can be experienced as tolerable or enjoyable, such as enjoying music as long as they are in control of the volume:

“I really like music and listen to it as much as I can during the day including during work and at all times on public transport and when shopping etc. I like being able to have full control of what I hear and how loud it is. I'm very specific in the music I listen to and will sometimes just listen to the same band/album/song for weeks...” SE022

#### Difficulty with Self-control

Several of the autistic adults also reported a difficulty with self-control when it comes to engaging with enjoyable sensory experiences. Although not all the autistic adults reported this as being a problem for them, some mentioned how this can be disruptive to their lives or a source of embarrassment:

“I can easily be completely distracted by certain decorative lighting; I can regularly spend long periods of time in the lighting section of a department store and find it difficult to leave.” SE046

“Love being able to touch soft and squashy things. Sometimes [I] have little control and [it] can be embarrassing when I do it inappropriately.” SE027

### The Role of Other People

Many of the autistic adults described how other people play a role in their sensory experiences. Other people were reported to be a source for self-understanding and making sense of the extent of sensory reactivity differences through comparison of their sensory experiences to other people. But also, sensory experiences can be impacted by other people’s understanding or misunderstanding of sensory reactivity differences. Furthermore, close relationships were also reported to be a source of support for autistic adults in relation to their sensory reactivity differences.

#### Understanding

The autistic adults frequently reported their own sensory experiences in comparison to what others experience. This was often in the context of comparing if their experiences of sensory input were more or less than others, or if their tolerance of sensory experiences better or worse than others, as a way of understanding if sensory reactivity was comparative rather than different to others. In some cases, these comparisons were based on their own perceptions of the behaviour of others, whereas others were based on information of sensory experiences provided by individuals:

“The lights in my dance studio flicker when first turned on. I close and cover my eyes to avoid this. Others don't seem to need to do this, though they note mild discomfort.” SE010

“When something happens where other people would feel pain I don't react as much and when people ask if everything [is] ok, I'm wondering if it should've hurt or not.” SE033

However, a few of the autistic adults reported how their sensory reactivity differences and their responses to sensory input may be misunderstood by others:

“At work the tube lighting is strong, so I go to the bathroom and keep the lights off for 5 min at a time… I think my colleagues probably assume I have a bowel issue. (I don't…)” SE015.

#### Support

Some of the autistic adults described how other people, especially significant relationships, were a source of support for sensory reactivity differences. They reported that others can help them navigate environments that they are finding challenging due sensory input, or can help them avoid discomfort or injury, such as if they are hyporeactive to pain or temperature:

“My partner will point out that I'm shivering before I've realised I'm feeling very cold. If my body is in pain it takes me time to realise, for example if my partner sits on my foot and it is in an awkward position, he'll notice, and I'll become aware it's painful once it has been pointed out.” SE15

Several of the adults also reported how other people were a source for supporting enjoyable sensory experiences, such as providing sensory input to help when feeling distressed:

“My [boyfriend] knows that if I have a meltdown the best help is to squish me as hard as he can until I feel ok again.” SE039

### Model of Sensory Reactivity Differences in Autistic Adults

**Fig. 3:**
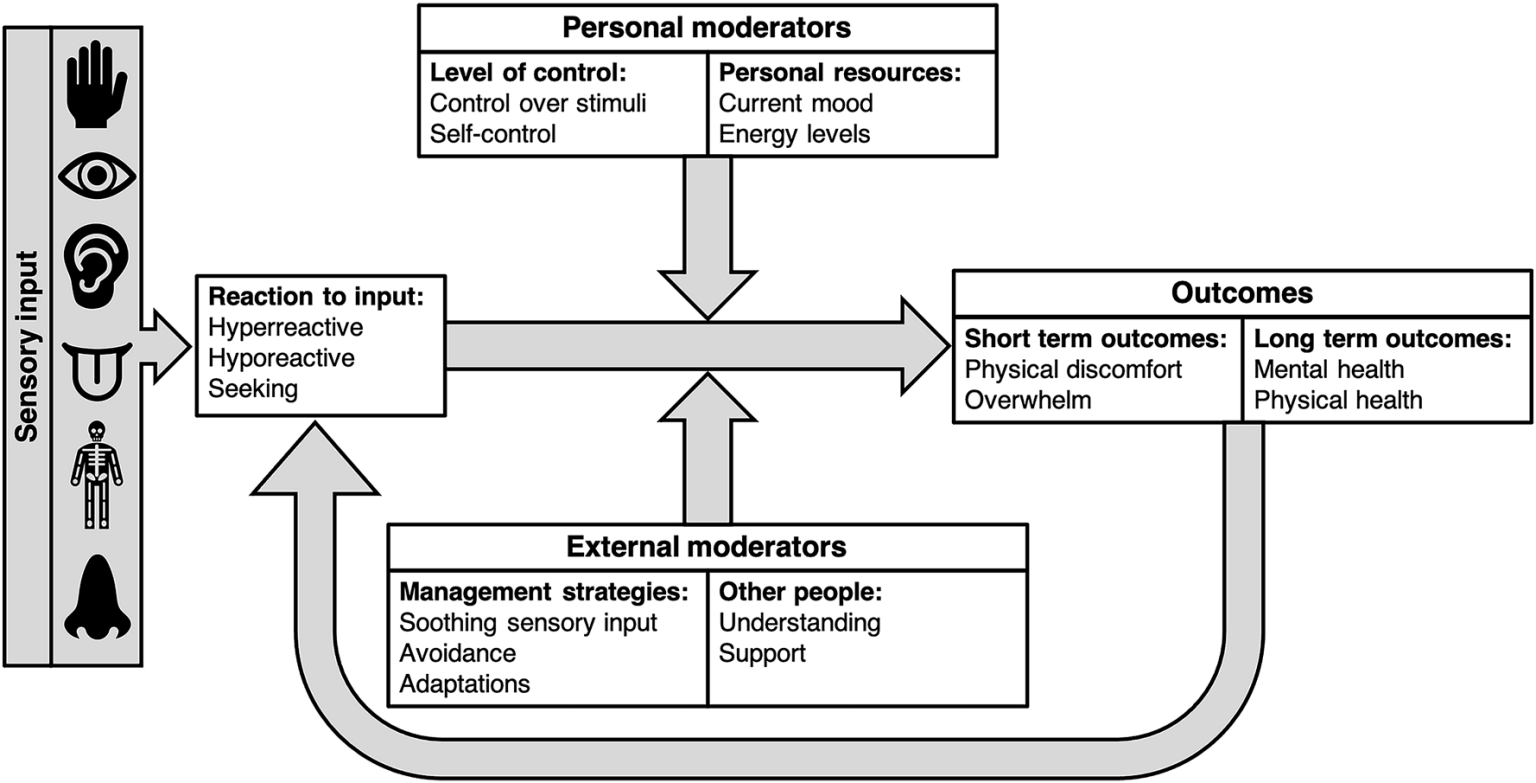
Summary of main themes and sub-themes developed from thematic analysis relating to sensory reactivity in autistic adults

Due to the interconnected nature of the themes and sub-themes developed in the thematic analysis, we propose a theoretical model depicting autistic adults’ experience of sensory reactivity differences (Fig. 3). We propose that reactivity differences to sensory input, hyperreactivity, hyporeactivity, or seeking, can have short-term outcomes, including experiencing physical discomfort or becoming overwhelmed or overloaded by input, as well as long-term outcomes for mental and physical health. These outcomes can then feedback to influence reactivity to sensory input. However, there are certain moderators that influence these outcomes for autistic adults, including both personal and external moderators. Personal moderators include the level of control over input and how predictable sensory input is, the level of self-control when engaging with enjoyable sensory input, and the level of personal resources, including current mood and energy levels. External moderators include management strategies, including avoiding and having the opportunity to escape sensory input, making adaptations to tolerate input, and regulating using soothing sensory input, and also the level of self-understanding, and understanding and support provided by other people.

## Discussion

This novel co-produced, mixed-methods study, has provided new and comprehensive insights into the complex sensory experiences of autistic adults. As well as elucidating stimuli and contexts associated with sensory hyperreactivity, hyporeactivity and seeking, across modalities, our results have enhanced understanding of autistic adults’ complex sensory experiences and informed the creation of a refined model from these experiences. Importantly, 96% of our sample identified as having sensory reactivity differences, supporting the persistence into adulthood (Crane et al., 2009), and highlighting the importance of researching sensory reactivity in autistic adults. Moreover, our results indicate that cooccurring sensory seeking and hyperreactivity (44.9%) was most experienced, whilst cooccurring sensory seeking and hyporeactivity was least experienced (0%). This supports that sensory seeking may be a strategy to regulate sensory input associated with hyperreactivity (Schulz & Stevenson, 2019), over being a stimulatory strategy associated with hyporeactivity.

### Sensory Input Related to Sensory Reactivity Differences

Our study adopted a novel mixed-methods approach to comprehensively outline sensory stimuli and contexts related to sensory reactivity differences in autistic adults. Although many of the sensory input identified, aligned with existing understanding, our results highlight the complexities of sensory reactivity differences across modalities, as well as the individualistic and contextual nature to sensory reactivity in autistic adults.

Our results echo existing research to indicate that sensory hyperreactivity is commonly experienced by autistic adults, with 93.3% of our sample self-identifying as being sensory hyperreactive. In line with previous research in autistic children and adults (Dickie et al., 2009; Robertson & Simmons, 2015; Schoen et al., 2008), we found that autistic adults experience hyperreactivity to a wide range of sensory input. However, our results importantly highlight that some sensory input commonly associated with sensory hyperreactivity are not endorsed by all individuals and are linked with personal preference, especially regarding colours and patterns, music, textures, tactile pressure, food tastes and textures, and scents, which individuals could be hyperreactive towards or seek out depending on their own personal preferences.

Our findings also provide a greater understanding of autistic adults’ experiences of sensory hyporeactivity. Sensory hyporeactivity was referred to less than other sensory experiences, with only 28.6% self-identifying as being sensory hyporeactive. Sensory hyporeactivity may become less prominent in adulthood, as sensory hyperreactivity and seeking increases (Liss et al., 2006), alternatively it may be underreported due to difficulties in self-reporting, as hyporeactivity is characterised by not noticing sensory input (Smith & Sharp, 2013). The autistic adults in our study described experiences of auditory hyporeactivity due to inattention. This suggests that hyporeactivity to sensory input may be due to inattention rather than neural processing differences, which could account for the variegated experiences of auditory hyper- and hyporeactivity experienced by an individual (Funabiki et al., 2012).

Lastly, our findings elucidated experiences associated with sensory seeking for autistic adults. Previous research has suggested sensory seeking may diminish with age (Crane et al., 2009; Kern et al., 2007), however, our results found 41.4% of the autistic adults to still engage in a diverse range of seeking behaviours. Specifically, our results provide deeper insight into the wide range of sensory input that autistic adults seek out across modalities. Sensory seeking was commonly individualistic, which is important to consider when using standardised sensory assessments that may not be accurately capturing the extent of an individual’s sensory seeking behaviours.

Overall, these findings have important implications for the development and interpretation of sensory assessments and the identification of support needs for autistic adults.

### Autistic Adults’ Experiences of Sensory Reactivity Differences

Our results also provide a greater understanding of autistic adults’ sensory experiences, a relatively under-researched area, and is the first known to understand sensory experiences related to all areas of sensory reactivity differences, including sensory hyperreactivity, hyporeactivity and seeking. Taking an iterative approach to thematic analysis, we developed themes relating to outcomes, the importance of control, tolerance and management, and the role of other people. Based on the interconnected nature of these themes, we propose a model of sensory reactivity experiences in autistic adults (Fig. 3). Although we are not the first to propose a model of sensory reactivity in autistic adults, and some of the findings are in line with these studies (see: Robertson & Simmons, 2015; Smith & Sharp, 2013), our mixed-methods approach and co-production with the autistic community has led to the development of some novel findings and a unique model, informed by our comprehensive results.

The autistic adults in our study described a range of outcomes related to their sensory reactivity differences. In our model, we propose that sensory reactivity differences can have short-term outcomes relating to physical responses and overwhelm. Our results importantly highlight autistic adults can become overwhelmed or overloaded due to sensory input and become disengaged with their circumstances or themselves. Sensory overload may result from hyper-focussing differences in autistic people, making it difficult to be able to divert attention away from aversive aspects of the sensory environment (Liss et al., 2006). This may contribute to ‘Burnout’, in which autistic individuals commonly experience chronic exhaustion and loss of skills, as well as experiencing heightened sensitivity to environmental stimuli and greater difficulty with tolerating or filtering out input (Raymaker et al., 2020).

Furthermore, our results suggest sensory reactivity differences have long-term outcomes relating to mental and physical health. Previous research has indicated that sensory hyperreactivity and hyporeactivity may be a risk factor for mental health conditions, such as anxiety in autistic adults (e.g., Hwang et al., 2019), and eating disorders in autistic women (Brede et al., 2020). However, our findings also suggest that sensory seeking, which is often considered to be an enjoyable experience, may also be associated with mental health conditions, as some autistic adults described links between their sensory seeking behaviours and self-harm and eating disorders. These findings are especially important as mental health conditions, such as anxiety, are disproportionately high in autistic populations (Buck et al., 2014). Moreover, our results highlight the physical impact of sensory reactivity differences. For instance, reduced food intake due to sensitivities towards food tastes or not noticing when hungry, can have consequences for physical health, or excessively listening to loud music can damage hearing.

Similarly to previous work (Robertson & Simmons, 2015), the autistic adults in our study described how they are more able to tolerate sensory input if they have control over the stimuli and it is less unpredictable. However, a novel finding in our study is that some of the autistic also described control in terms of their difficulties with self-control, such as finding it hard to disengage with certain enjoyable sensory input. Sensory reactivity differences are part of the diagnostic criterion for autism associated with restricted and repetitive behaviours (DSM-5 American Psychiatric Association, 2013), and research has found sensory seeking to relate to ritualistic/sameness behaviours in autistic children (Boyd et al., 2010). Therefore, our findings suggest these behaviours persist into adulthood.

Although various coping mechanisms and strategies for sensory reactivity differences have previously been shown in qualitative research (Robertson & Simmons, 2015; Smith & Sharp, 2013), we found avoidance, making adaptations, and engaging in soothing sensory strategies to be commonly used coping mechanisms/strategies. The autistic adults in our study emphasised the importance of being able to escape from unbearable sensory input to avoid sensory overload. Avoidance is often considered a maladaptive strategy for anxiety, due to avoidance impacting the ability to regulate arousal, which then increases and maintains anxiety, leading to more avoidance (Green et al., 2012; Joosten & Bundy, 2010; Lidstone et al., 2014; Mazurek et al., 2013). However, it may be an essential strategy for autistic individuals. Our results provide additional insights into the extensive use of adaptive strategies used by autistic adults, such as using earplugs/headphones to tolerate noisy environments, or sunglasses in bright environments. Furthermore, the autistic adults in our study also described how they engage with soothing sensory input as a regulation strategy. Our findings suggest that autistic adults also sensory seek across modalities, engaging with personally preferred scents, textures, and visuals that are experienced as soothing and enjoyable. Additionally, in line with previous work (Robertson & Simmons, 2015; Smith & Sharp, 2013), our results show listening to chosen music is a common strategy found to be soothing. Music has been identified as a common interest of autistic adults, and engagement with significant or ‘special’ interests is related to greater subjective wellbeing (Grove et al., 2018).

### Limitations and Future Directions

Although a key strength of our study was the online survey approach, facilitating the potential for a diverse range of perspectives and experiences from geographically dispersed populations, online research has some potential limitations. For instance, we were unable to provide support for individuals who may have struggled with the open-ended questions within the questionnaire. Although we encouraged respondents to seek support, this may have restricted participation in this study, especially for those without access to support, or individuals with higher support needs or intellectual disability (ID). Although 4% of our participants reported to have cooccurring ID, this is not representative of the autistic population, within which around 50–55% have cooccurring ID (Charman et al., 2011; Loomes et al., 2017). Autism research often underrepresents individuals with ID (Russell et al., 2019), and therefore future research should endeavour to understand the sensory experiences of these individuals.

## Conclusion

Our study demonstrates that sensory reactivity differences are prominent in autistic adults, and that they are complex, individual, interconnected, and experienced across a range of modalities. We propose a theoretical model of sensory reactivity differences informed by the experiences of autistic adults. Crucially, it highlights key moderating factors that may reduce the long-term impact of sensory reactivity differences on physical and mental health. Future work could be informed by our model and aim to understand more about the positive and negative impacts of the identified moderators of sensory reactivity differences. Our findings have important implications for support services and future research that aims to reduce the impact of sensory reactivity differences for autistic adults.

## Supplementary Information

Below is the link to the electronic supplementary material.

Supplementary file1 (DOCX 15 kb)

## Footnotes

1 The terms used in this paper are based on the preferred terms that have been outlined by the autistic community (Kenny et al., 2016).
