# Supplementary material for: In Our Own Words: The Complex Sensory Experiences of Autistic Adults
Source: J Autism Dev Disord. 2021 Jul 13;52(7):3061–75. doi: 10.1007/s10803-021-05186-3 (PMC9213348; doi:10.1007/s10803-021-05186-3)
Supplement: Supplementary file 1 — Supplementary file1 (DOCX 15 kb) [file 10803_2021_5186_MOESM1_ESM.docx]

**Supplementary table 1**

Example quotes from the content analysis for experiences relating to sensory hyperreactivity, hyporeactivity, and seeking, and in each modality (n = 49).

|  | ***Hyperreactive*** | ***Hyporeactive*** | ***Seeking*** |
| --- | --- | --- | --- |
| *Visual* | “Sunlight is overwhelming in the summer and I can't stand being out for too long... I also can't stand bright overhead lighting in shops and tend to leave as quickly as possible. Lower overhead lighting is worse and fluorescent is unbearable…” SE029 | “There are numerous occasions where I was looking for an item which was in plain sight however I it took a long time to find it.” SE003 | “I can get lost in visual patterns. When I was little one of the churches, we sometimes attended had a patterned coving and I would spend the whole service visually following the infinite line that formed the pattern.” SE010 |
|  |  |  |  |
|  | “A busy visual field causes overload and causes agitation and stimming. I struggle to focus on specific components and take in the entire field, being aware of every movement.” SE016 |  |  |
| *Auditory* | “With multiple conversations… my experience is similar to listening to a radio station that then blends into the other one, so you're constantly hearing every other word of the radio station, until at some point the streams completely overlap, but somehow in complete clarity. This, along with all the loud environmental noise (which may seem like nothing to most people - air conditioning humming, projector buzzing, lights buzzing, plates clinking in a restaurant kitchen), can drive me into a shutdown. And then every sound is utterly overwhelming.” SE040 | “Some sounds make me sleepy and if I'm sleepy or focused I just won't hear things. Like I was reading one time and didn't hear the fire alarm.” SE039 | “Music calms me down when I'm stressed. Tend to listen to the same song on repeat for hours at a time.” SE022 |
| *Tactile* | “I have to check the texture of fabrics when I'm clothes shopping. Anything rough or crunchy I can't wear. I also need soft and stretchy fabrics.” SE038  “I don’t like unexpected touch from other people. I prefer firm touch to light touch.” SE011  “I’m touch sensitive. I hate other people touching me. My skin crawls, I sweat excessively, and I feel nausea. Especially if it’s bare skin. Makes intimacy nigh on impossible.” SE030 |  | “It is extremely difficult to provide me with enough, let alone too much, deep pressure. Sometimes I will ask my husband for a tight hug and even the tightest hug he can provide is insufficient to satisfy me. Often, I need him to lie on top of me to provide added pressure.” SE046  “Fluffy textures calm me. I have a fluffy blanket in the car to help manage my anxiety on car journeys and one I carry in my bag to calm me on other occasions.” SE016 |
| *Interoception* | “Heat can be utterly unbearable, especially because it interferes with some of my other sensory preferences such as pressure seeking. Even in ordinary warm summer days around 20C, my functioning is impaired, and I feel discomfort. During a true ‘heatwave’ I can be essentially in a permanent state of shutdown. This sensitivity extends to hot water, as I have an immediate recoil tendency upon entering water even approaching scalding hot.” SE046 | “I have failed to notice broken bones. I went hiking the day after breaking my foot and walked to school the day after focally fracturing my shin.” SE024  “I don't notice until 'too late' that I'm too cold [or] too hot” SE015 | “In cold weather I make a blanket nest and a hot water bottle on my back and on my stomach.” SE030 |
| *Gustatory* | “I am very particular about tastes. I only like quite bland foods and can’t stand any kind of spice. I will find a food unbearably spicy that others say has no spice to it at all. I used to find fizzy drinks too intense when I was younger.” SE011 |  | “I will continuously seek tastes that I enjoy.” SE003  “I eat when I'm not hungry because I want the taste.” SE019 |
| *Olfactory* | “I have had to quit jobs and refuse assignments due to my being hyperreactive to scents… My aversion to strong, unpleasant scents is so strong that it triggers my gag reflex, can make me throw up, makes me cry, and makes me escape the environment. I have tried but have no control over it. Changing nappies for my kids was challenging. Usually my husband did it. If my kids vomited, the smell made me vomit.” SE031 | “Often I can't smell subtle smells like flowers or smoke that other people comment on.” SE022 | “I have a jar of cinnamon in my soothe box, and sawdust because that evokes my pets and makes me calmer… I smell my hands a lot without thinking about it - sometimes it is annoying when they smell of soap. I like it when they smell of yesterday's onions I was chopping.” SE10 |
|  |  |  |  |
